# MIMO-enhanced talkative power conversion: a perspective

**DOI:** 10.1038/s44172-026-00720-6

**Published:** 2026-07-10

**Authors:** Peter A. Hoeher, Yang Leng, Rongwu Zhu

**Affiliations:** 1 Faculty of Engineering, Kiel University, 24143 Kiel, Schleswig-Holstein Germany; 2 Department of Electrical Engineering, Harbin Institute of Technology, Shenzhen, Guangdong China

**Keywords:** Electrical and electronic engineering, Power distribution

## Abstract

Talkative Power Conversion (TPC) is an instance of simultaneous information and power transfer, in which data modulation is integrated in the power conversion process. The TPC framework is generalized in this work to incorporate multiple-input multiple-output (MIMO) processing. In this innovative concept, called MIMO-TPC, power electronic converters with multiple switching units are applied that use coordinated or uncoordinated, data-dependent switching patterns in order to increase the degrees of freedom for data modulation. A selection of power converter topologies is investigated that fit within the framework of MIMO-TPC, including H-bridge DC/DC converters, multi-phase DC/DC converters, multi-phase DC/AC inverters, multi-input converters, multi-modular converters, multi-level converters, and multi-port converters. These switched-mode converter classes are characterized by an elementary building block and elementary combinations. Encouraging advantages of MIMO-TPC are elaborated, potential applications of MIMO-TPC are discussed, and open challenges as well as future research directions are suggested.

## Abstract (web summary)

Talkative Power Conversion (TPC) integrates data modulation directly into power converters. Peter A. Hoeher and colleagues explore a generalization called MIMO-TPC, which uses coordinated switches to create additional pathways for embedding data within the converters.

## Introduction

In systems theory, the term multiple-input multiple-output (MIMO) denotes a system with multiple inputs and outputs, see for example ref. ^1^. MIMO signal processing has a wide range of applications. All these applications have multi-port systems and vector-valued signals in common.

Radio systems: In wireless radio, MIMO refers to a system with multiple antennas, both on the transmitter and the receiver side, for the purpose of improving the performance of the communication channel^2–5^. MIMO systems can transmit more bits per second per Hertz bandwidth and therefore have a higher spectral efficiency compared to conventional single-input single-output (SISO) systems with one antenna on both sides^6,7^. Special cases of MIMO systems are single-input multiple-output (SIMO) and multiple-input single-output (MISO) systems, respectively^8^. Adaptive antenna arrays, so-called smart antennas, can be used for beamforming and precoding^9^, throughput enhancement (called spatial multiplexing^10^), and/or outage reduction (called spatial diversity^11,12^). Antenna arrays with hundreds of antenna elements are called large-scale MIMO or massive MIMO systems^5,13,14^. Massive MIMO can also be realized with multi-mode multiport antennas^15,16^, enabling ultra-high-speed communications and massive connectivity^17,18^. With spatially distributed antennas, outage can be reduced by diversity combining^8,19^. MIMO processing is used in Wi-Fi and 3 G/4 G/5 G cellular radio systems^20^. Beside communication purposes, with MIMO radio systems localization is possible^21^. Swarm scenarios can also be modeled as spatially distributed MIMO systems^22^.

Radar systems: In MIMO radar, antenna arrays are used for adaptive beamforming, to increase the effective aperture size, to improve the spatial resolution, to reduce radiation lobes, and to achieve better immunity against interference^23,24^. There are two classes of MIMO radar: multistatic radar, also known as statistical MIMO radar, and coherent MIMO radar. One advantage of coherent MIMO radar signal processing is the ability to increase the angular resolution of the physical antenna array by forming a virtual array^24^.

Sonar systems: In sonar systems, sound transducers are replacing radio-frequency antennas. MIMO-based sonar systems offer perspectives for acoustic underwater communication, high-resolution underwater imaging, target detection, and underwater surveillance, among other use cases^25,26^.

Wireless communication systems: Communication cables that consist of twisted wire pair bundles are characterized by cross-talk between the wires. The input/output behavior of cable bundles can be modeled as a MIMO system^27^. With MIMO signal processing, the data rate can be boosted noticeably^28^. Similarly, three-phase power line communication (PLC) systems can be modeled as MIMO scenarios^29^. The data rate can be increased by exploiting all three phases. Besides these communication-related applications, multiport converters have been treated in the power electronics community as MIMO converters^30–32^. A MIMO converter can route the power flow between its multiple input and output ports, but so far, no data transfer is considered.

Control theory: In control theory, MIMO systems are frequently described by a state-space model. Among the challenges are optimal control of MIMO systems and system stability in time-varying scenarios^33^. For MIMO systems, the controller design is more complicated than for SISO systems.

The aim of this paper is to apply MIMO processing to another field, namely, simultaneous information and power transfer. Simultaneous information and power transfer is an emerging topic with applications encountered in very different areas, including electric grid applications, wireless information and power transfer, distributed control, battery management systems, motor drive control systems, routers, etc.^34^. Data modulation and power transmission can be done either separately or together. PLC and Power-over-Ethernet (PoE) are examples of the first solution, because the data stream is inserted onto the power line via a coupler^29^ or a PoE splitter^35^, respectively. Consequently, an auxiliary modulator/demodulator is needed. In contrast, this paper targets joint data modulation and power transmission on the basis of talkative power conversion (TPC), an expression that was created in ref. ^36^ based on the terms Power Talk^37,38^ (targeting system control of networked power converters by communication signal injection in the control loop) and Talkative Power^39^ (targeting the physical layer of communication-enhanced power conversion using switching ripple modulation). TPC is a technique in which data modulation is integrated in the power conversion process, leading to simultaneous information transmission and power conversion, while considering all layers of the Open Systems Interconnection (OSI) reference model^40^. Consequently, the roots of TPC are in power electronics (PE) and communications (COM). As opposed to PLC and PoE, no or few additional transmitter hardware is necessary for simultaneous information and power transfer^41^. Therefore, system reliability and real-time performance are improved, while system complexity, cost and volume are reduced.

**Fig. 1:**
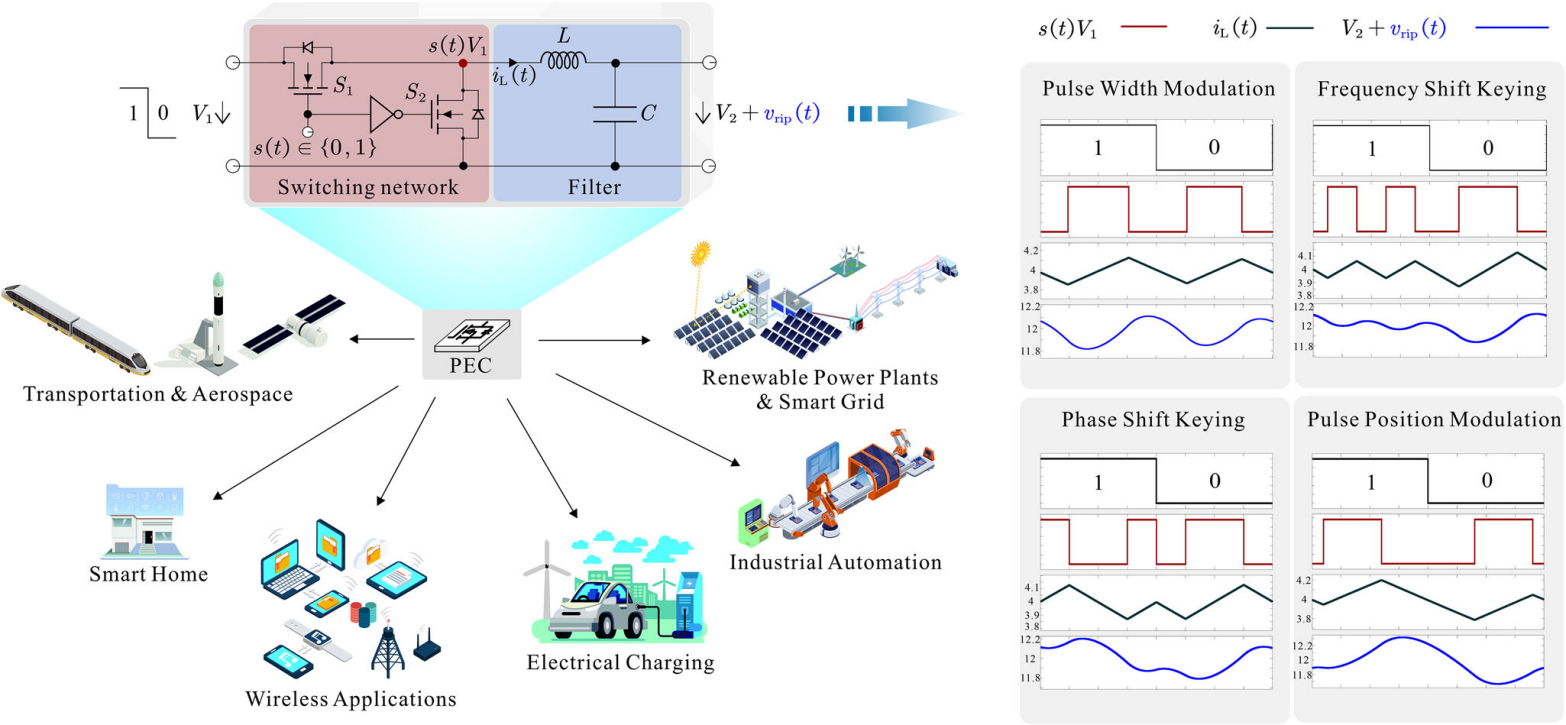
Synchronous buck converter as an example of SISO DC/DC power converter topologies and potential applications of TPC. The power electronic converter is the central device, as it is able to simultaneously adjust power flow and perform information transfer with almost no extra hardware.

The central element of TPC is a switched-mode power electronic converter (PEC). PECs serve as high-efficient step-down and/or step-up converters and are popular in wireline as well as wireless applications (Fig. 1). In the top part of this figure, a synchronous buck converter is illustrated as an example. Given a DC input voltage *V*_1_, in a first step, a pulse-width modulated voltage (depicted in red color) is generated by a switching network. The pulse width/frequency/position/phase is modulated for information transmission. In a second step, this modulated square-wave signal is averaged, typically by an LC low-pass filter. From a PE point of view, the average DC output voltage *V*_2_ is of primary interest, while the ripple voltage must satisfy electric power quality constraints^42^. Vice versa, from a COM point of view, the information must be extractable from the ripple voltage even in noisy environments.

In the bottom part of the figure, a selection of possible TPC applications is listed. These include transportation and aerospace applications (e.g., on-board data bus systems in cars, busses, trains, aircraft, and aeronautics), smart home applications (e.g., Internet of Things, infotainment, and home automation), wireless applications (e.g., wireless sensor networks and wearables), electrical charging (e.g., of medical implants, consumer electronics, and e-vehicles, robots), industrial automation (e.g., automated guided vehicles and industrial automation machinery), renewable power plants (e.g. energy flow optimization) and smart grid applications (e.g. grid control, smart metering, and electric energy storage), among others. For further details, interested readers are referred to the overview papers like refs. ^36,39,40,43^ and references therein. In summary, TPC is characterized by low cost, low latency, and a wide range of possible applications that go far beyond PLC. Among the disadvantages of TPC are a small data rate and a limited communication range, both according to the state-of-the-art. We should also keep in mind that, at least in grid applications, the simplicity and efficiency of power conversion are of primary interest, while communication usually plays a secondary role.

The history of TPC goes back to ref. ^44^, where the switching signal of a switched-mode power supply was digitally modulated. This way, the switched-mode power supply jointly serves as a data transmitter and a voltage transformer. The information is embedded in the ripple voltage, which superimposes on the output voltage of the converter. Hence, TPC is an instance of switching ripple modulation^45^. In recent publications, additional types of power converters^41,46–55^, various modulation schemes^56–63^, coding aspects^58,64^, multiuser aspects^37,38^, voltage control^65,66^, networking aspects^67–69^, and a variety of applications^65,66,70–79^ have been investigated. Also, surveys and tutorials^36,39,40,43^ have recently been published. Other names include power/signal dual modulation^46,56^, switching ripple communication^64^, power and signal synchronous transmission^43^, and zero-additional-hardware PLC^41^.

In this Perspective, the TPC framework is extended to incorporate MIMO signal processing, a concept which is dubbed MIMO-TPC. MIMO-TPC is explained for selected power converter topologies using an elementary building block and elementary combinations. The data-dependent ripple voltage and the inductor current are numerically calculated for the selected power converter topologies. Numerous research questions considering MIMO-TPC evolutions are suggested.

MIMO signal processing is expected to partly reduce the bottlenecks of conventional TPC designs. Additional MIMO-TPC techniques and an in-depth analysis are stipulated for future work. Generally speaking, potential use cases of MIMO-TPC are expected to be in the same areas as conventional TPC. However, by means of MIMO processing, more sophisticated applications/opportunities are anticipated. For example, new consumer products are expected if higher data rates are offered, and larger communication distances and applications in harsh environments are likely if more robustness is provided.

## Framework from SISO-TPC to MIMO-TPC

### SISO-TPC techniques

TPC is applicable to all switched-mode power converter topologies. A simple example is the synchronous buck converter (Fig. 1), which is a popular DC/DC step-down converter. A buck converter consists of a half-bridge (with switches *S*_1_ and *S*_2_) followed by a low-pass filter. The half-bridge is driven by a single pulse-width modulated switching signal *s*(*t*) with duty cycle *D* ∈ (0, 1). The task of the low-pass filter, here a second-order LC filter, is to smooth the square waveform produced by the half-bridge and to reduce undesired radiation. Assuming a fixed input voltage *v*_1_(*t*) = *V*_1_, the output voltage *v*_2_(*t*) = *V*_2_ +* v*_rip_(*t*) can be decomposed into a mean output voltage, *V*_2_, and a time-varying ripple voltage, *v*_rip_(*t*). In the steady state, *V*_2_ = *DV*_1_ holds for ideal switches. In electric grid applications, the ripple voltage should be below a certain threshold to ensure electric power quality^42^. Regarding TPC, on the other hand, the message is included in the ripple voltage. Hence, there is a trade-off between PE and COM requirements. This limits the data rate and communication distance. The vulnerability of weak information-carrying signals against noise and interference can be partly solved by repetitions and channel coding^58^. Another drawback of SISO-TPC techniques is that, due to the single two-level switching signal, the degrees of freedom for data modulation are limited: only the pulse width, the pulse frequency, the pulse position, and/or the pulse phase can be modified^58,62^. Correspondingly, there are four elementary modulation schemes in this context: PWM (pulse width modulation), FSK (frequency shift keying), PPM (pulse position modulation) and PSK (phase shift keying), as well as combinations thereof. Among the motivations of this paper is to increase the number of degrees of freedom by MIMO-TPC. For example, with two half-bridges, ASK (amplitude shift keying) can be realized. The half-bridge is the elementary building block under investigation.

In ref. ^58^ and ref. ^80^, the sampled output voltage v_2_[*n*] = *V*_2_ + *v*_rip_[*n*] of a synchronous buck converter (which always operates in continuous conduction mode by design) and the inductor current *i*_L_[*n*] were computed numerically for an arbitrary switching signal *s*[*n*], a constant input voltage *V*_1_ or a time-varying input voltage *v*_1_[*n*], and an Ohmic load *R*_L_:

1 $${i}_{{{\rm{L}}}}[n]={i}_{{{\rm{L}}}}[n-1]+\frac{\Delta t}{L}\underbrace{\left(\underbrace{s[n-1]{V}_{1}}_{{v}_{1}[n-1]}-{v}_{2}[n-1]\right)}_{{v}_{{{\rm{L}}}}[n-1]}$$

2 $${v}_{2}[n]={v}_{2}[n-1]+\frac{\Delta t}{C}\underbrace{\left({i}_{{{\rm{L}}}}[n-1]-\underbrace{\frac{{v}_{2}[n-1]}{{R}_{{{\rm{L}}}}}}_{{i}_{{{\rm{R}}}}[n-1]}\right)}_{{i}_{{{\rm{C}}}}[n-1]},$$

where *n* is the time index and ∆*t* =* T*_sw_*/J* is the time step with oversampling factor *J*, i.e., *v*_*x*_[*n*] = *v*_*x*_(*t* = *n*∆*t*) with *x*∈{1, 2} and *i*_*L*_[*n*] = *i*_*L*_(*t* = *n*∆*t*). As shown in ref. ^80^, any sufficiently large number of samples per switching period (e.g., *J* = 100) causes a negligible waveform modeling error in simulations. The output voltage comprises the DC plus ripple components. The recurrence relations result from Kirchhoff’s laws and are calculated alternately. The initial conditions are *i*_L_[0] and *v*_2_[0], respectively. According to Eq. (1), the inductor current increases linearly with the voltage across the inductor, while according to Eq. (2), the voltage across the capacitor increases linearly with the current through the capacitor. If the time step ∆*t* is chosen to be much shorter than the switching period *T*_sw_, i.e., if the oversampling factor *J* is sufficiently large, the numerical solution converges to the true output voltage. The recurrence relations are applicable for the steady state as well as the transient behavior and hold for arbitrary data sequences. For the buck converter and related SISO architectures, in the case of ideal switches, *s*[*n*] has only two levels and can be normalized as *s*[*n*]∈{0, 1}. As will be examined in subsequent sections, Eqs. (1) and (2) are still valid for MIMO architectures with multiple levels. Furthermore, it is interesting to note that Eqs. (1) and (2) hold for arbitrary pulse shapes as well (which are inherently embedded in the series of samples *s*[*n*]), and can therefore be used in particular for switches with finite switching times. The relation between *D* and *s*[*n*] is given by *D* = E{*s*[*n*]}, where E{.} denotes the expected value operator.

Equations (1) and (2) (in combination with Eqs. (3)-(6)) supplement and strengthen the conceptual approach under investigation. These equations are relevant as the waveforms can be utilized to compute parameters such as peak inductor current and total harmonic distortion. This knowledge is important for circuit design. These equations also form the basis for optimal receiver structures such as maximum-likelihood detection^58^.

### MIMO-TPC techniques

In this section, we generalize the TPC framework to incorporate MIMO processing. In this concept, called MIMO-TPC, power electronic converters with multiple switching units are applied that use coordinated or uncoordinated, data-dependent switching patterns in order to increase the degrees of freedom for data modulation^40^. In particular, we highlight a selection of power converter topologies that fit within the framework of MIMO-TPC. Suitable converter topologies include H-bridge DC/DC converters, multi-phase DC/DC converters, multi-phase DC/AC inverters, multi-input converters, multi-modular converters, multi-level converters, and multiport converters. In this paper, the term “MI” in “MIMO” is assigned to multiple data input ports. These are able to act as multiple transmitters (like the multiple transmit antennas in MIMO radio systems), as each port can be assigned a data-dependent switching pattern. Likewise, the term “MO” is assigned to multiple data output ports, i.e., to different receiver ports. Receiver ports could be, for instance, receiver-side voltage or current sensors providing multiple measurements (like the multiple receive antennas in MIMO radio systems). In analogy to mobile radio, the term “MIMO” is used as a generic term here and also includes SIMO and MISO scenarios.

Although in this paper “MIMO” is related to the direction of the information flow, “MI” and “MO” can alternatively be assigned to the power flow. In the PE community, “MIMO” is naturally associated with the power flow, especially in conjunction with multiport converters^30,31^. In the proposed MIMO-TPC concept, however, information and power flows can even go in opposite directions—data input ports and power input ports (respectively data output ports and power output ports) do not necessarily need to be identical^55^. Furthermore, the concept proposal is more generally applicable than just to multiport converters.

The availability of multiple transmitter ports allows for the generation of coordinated or uncoordinated switching patterns. This facilitates various options for multi-bit transmission. One option is to increase the cardinality of the symbol alphabet—similar to a higher-order modulation scheme. For instance, the number of ripple voltage levels could be increased. This can be achieved with a single full bridge as well as with two-phase DC/DC converters, multi-input, multi-level, and multi-modular converters, among other topologies. A single receiver port is adequate for this purpose, yielding a MISO configuration. Another approach is to transmit different data sequences through different ports. This method is akin to spatial multiplexing used in wireless communication systems. In this MIMO scenario, the number of receiver ports must not be less than the number of independent data sequences in order to avoid ambiguities in the data detection process. This can be accomplished in conjunction with a multi-phase DC/AC inverter or a multiport converter, for example. A SIMO configuration can be exemplified by combining decisions from multiple spatially distributed receivers that share the same bus and observe the same transmit signal. This approach is related to the concept of spatial diversity commonly applied in wireless radio. Potential applications include noisy environments or TPC systems using a low ripple voltage.

Sophisticated topologies can be derived by using the half-bridge as an elementary building block in conjunction with elementary combinations: parallel combination, interleaved combination, series combination, and cascaded combination. These combinations can be applied to the half-bridge and/or to modules that typically comprise several half-bridges. A similar concept was presented in ref. ^81^, but with different objectives. In ref. ^81^, a voltage-source generalized multi-level converter concept was proposed that unifies several generalized multi-level topologies. Known as well as new multi-level topologies can be derived from this concept. This helps to understand complicated converter topologies. However, the focus of the work in this paper is on the degrees of freedom for data transmission that this concept offers. The existence of further building blocks and/or combinations is not excluded. For didactic reasons, emphasis will be on a few common topologies.

### H-bridge DC/DC converters

**Fig. 2:**
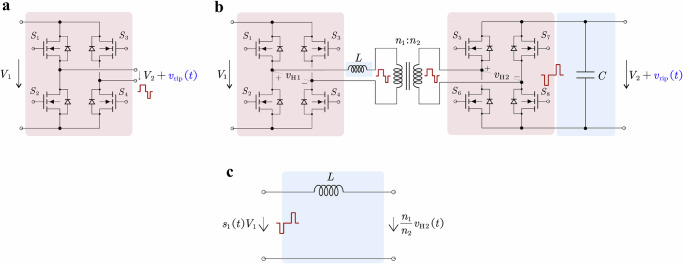
H-bridge-based DC/DC converters. **a** Full bridge. **b** Dual-active bridge converter. **c** Equivalent model of **b** assuming an ideal transformer.

A full bridge, commonly referred to as an H-bridge, is formed by two half bridges in parallel (Fig. 2a). A parallel combination is the first elementary half-bridge combination under investigation. Isolated or non-isolated DC/DC power converter topologies that employ at least one full bridge are subsequently called H-bridge DC/DC converters. Every leg is controlled by a PWM-type switching signal. Having multiple switching signals available potentially increases the degrees of freedom for data transmission.

A single H-bridge can produce a three-level output voltage: (i) The voltage is positive if switches *S*_1_ and *S*_4_ are ON; (ii) The voltage is reversed if switches *S*_2_ and *S*_3_ are ON; (iii) The voltage is zero if switches *S*_1_ and *S*_3_ are ON or *S*_2_ and *S*_4_ are ON. Therefore, compared to a half-bridge, the cardinality of the symbol alphabet is increased in the context of TPC.

A prominent example of converters that use a full bridge is the dual-active bridge (DAB) converter^82^. This topology uses two H-bridges that are typically separated by a transformer plus a series inductor. The first H-bridge performs DC/AC conversion, while the second H-bridge performs active AC/DC rectification. Capacitors are connected in parallel to the DC input and DC output. This topology supports bidirectional power transfer. Popular power modulation schemes applied to DAB converters are single phase shift, extended phase shift, dual phase shift, and triple phase shift control^83,84^. A generalization thereof is 5-degree-of-freedom (5-DoF) modulation, treating popular power modulation schemes as special cases^85^. The 5-DoF modulation scheme can be seen as a combination of two independent switching patterns for each H-bridge, hence its TPC extension proposed in ref. ^55^ fits well within the MIMO-TPC concept.

DAB converters are currently under investigation, for instance, for on-board chargers in automotive applications. Although DAB converters are widely used in industry, so far, there are only a few journal publications that apply TPC to this topology. In ref. ^47^, the soft-switching capabilities of the DAB converter were enhanced by applying a switching frequency variation strategy. In ref. ^86^, a transient performance analysis of a TPC-based DAB converter was performed for PWM-FSK and PWM-PSK modulation. Power and communication decoupling for a DAB converter with FSK-based TPC has been investigated in ref. ^54^. In ref. ^55^, seamless embedding communication into the family of H-bridge-based multiport power converters has recently been done by data-modulating the DoFs used for power control. In the same article, the following recurrence relations have been derived for the special case of a DAB converter:

3 $${i}_{{{\rm{L}}}}[n]={i}_{{{\rm{L}}}}[n-1]+\frac{\Delta t}{L}\left(\underbrace{{s}_{1}[n-1]{V}_{1}}_{{v}_{{{\rm{H1}}}}[n-1]}-\underbrace{{s}_{2}[n-1]{v}_{2}[n-1]}_{{v}_{{{\rm{H2}}}}[n-1]}\right)$$

4 $${v}_{2}[n]={v}_{2}[n-1]+\frac{\Delta t}{C}({s}_{2}[n-1]{i}_{{{\rm{L}}}}[n-1]-{v}_{2}[n-1]/R),$$

where *v*_H1_ and *v*_H2_ are the bridge voltages of the H-bridges depicted in Fig. 2b. A 1:1 transformer winding ratio is assumed here for simplicity.

Another example of converters that use a full bridge is the phase shift full-bridge converter^87^. This converter consists of an H-bridge and a passive rectifier. Frequently, the diode-based rectifier is separated from the H-bridge by a transformer plus a series inductor. The ripple voltage can be adjusted by a phase shift between the two legs of the H-bridge^46^. Accordingly, the system can be adopted to different operating conditions.

### Two-phase Interleaved DC/DC converters

**Fig. 3:**
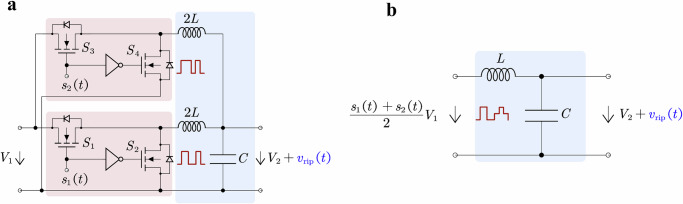
Two-phase interleaved buck converter. This topology consists of interleaved half-bridges and is suitable to realize quadrature amplitude modulation (QAM) signal constellations. **a** Topology. **b** Equivalent model of (**a**).

Modulation schemes with a real-valued symbol alphabet are considered in the majority of TPC publications. However, complex-valued modulation schemes are known to increase spectral efficiency^88^. It is possible to generate data symbols with independently adjustable amplitude and phase by employing a two-phase power converter (Fig. 3a), also called a two-leg interleaved synchronous buck converter. This technique is capable to achieve up to 3 bps/Hz switching frequency utilization with 64-QAM and up to 4 bps/Hz with 256-QAM^70^. These spectral efficiencies are the highest that have ever been documented for TPC.

The versatility of interleaved converters is illustrated by the fact that they can also generate multi-carrier modulation (MCM) schemes^74^. Orthogonal frequency-division multiplexing^88^ is a well-known example of MCM^60,61^. A two-phase interleaved buck converter can be used to determine the amplitude and phase of an MCM communication signal. Recently, ref. ^78^ proposed a novel variation of switching ripple modulation in which a single-phase buck converter was used to realize QAM constellations^88^. This is possible if *D <* 0.5. Then, groups of two consecutive pulses can be modulated.

Interleaving is the second elementary combination under investigation. The ability of the two-phase interleaved buck converter to realize complex-valued symbol constellations is based on two principles: (i) A sinusoidal signal whose amplitude and phase can be set independently of each other can be generated by superimposing two sinusoidal signals with the same amplitude and frequency by changing their phases^70^; (ii) The desired switching signal is the first harmonic of the superimposed sinusoidal signal. The 2-level switching signal can be either obtained from the superimposed sinusoidal signal by using a comparator, or can be directly delivered by a microcontroller, for example, using a look-up table.

These two principles are exploited next for two sinusoidal signal *c*_x_(*t*) with equal amplitude and frequency *f*_sw_ but different phases *γ*_x_, where *x*∈ {1, 2}. The amplitude and the phase of their sum *c*(*t*)=*c*_1_(*t*)+*c*_2_(*t*) can be controlled independently. Hence, *M*-ASK, *M*-PSK and *M*-QAM are realizable with arbitrary cardinality *M* in two steps. According to ref. ^70^, given *M* and the desired symbol constellation, classically, a set of *M* pairs [γ_1_, γ_1_] is first (pre-)calculated and stored. The two waveforms *c*_x_(*t*) are then converted into data-dependent PWM signals with a fixed duty cycle *D*^89^: *s*_x_(*t*) = rect(*c*_x_(*t*)), where *s*_x_(*t*) = 1 of *c*_x_(*t*) is larger than a duty cycle-dependent threshold and zero else. These square waveforms serve as switching signals, i.e., these are the input signals of the two-phase buck converter (Fig. 3a).

Owing to its current-handling capability and low ripple voltage, the interleaved topology can be used, for example, for servers in data centers, radio base stations, precision instruments, and medical devices. It is interesting to note that the output voltage of the two-phase buck converter can be computed by means of Eq. (2) in conjunction with Eq. (1), where *s*[*n*] ∈ {0, 0.5, 1} is a sampled version of the sum of the two PWM signals. The equivalent model is depicted in Fig. 3b.

### Multi-phase DC/AC inverters

The majority of TPC research has focused on DC/DC converters thus far; however, ref. ^37^ already predicted that TPC would eventually be extended to AC microgrid systems. Initial research has been published in ref. ^90^. Three-phase DC/AC conversion methods based on space vector PWM were recently proposed in refs. ^49^ and ^53^. In a switching cycle, the spatial vector distribution has varying degrees of freedom. This method can be divided into two categories: variable zero-vector position-based modulation and variable zero-vector width-based modulation, depending on the width and position of the zero-vector.

**Fig. 4:**
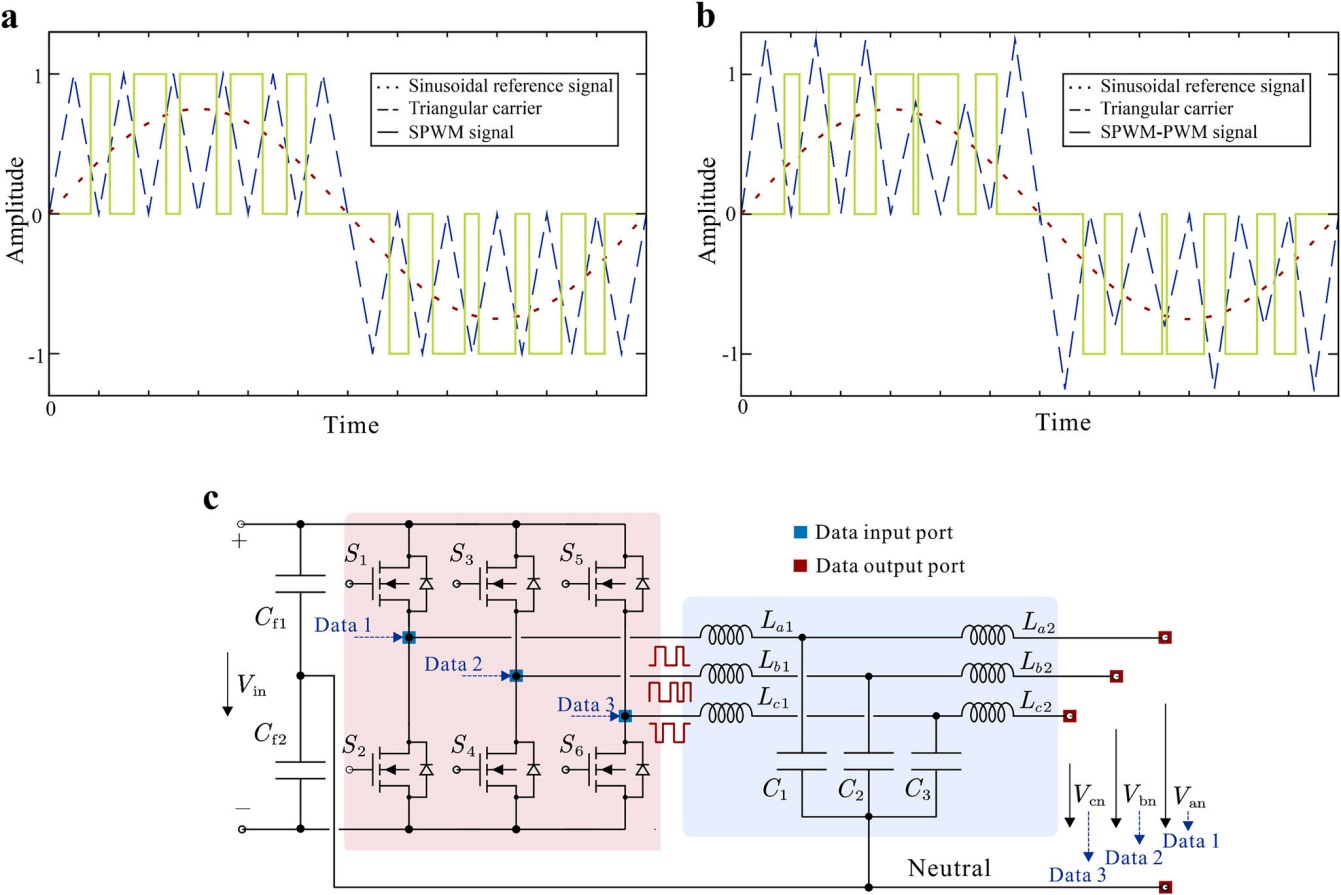
Sinusoidal pulse width modulation (SPWM). **a** Periodic SPWM waveform. The switching frequency is twelve times larger than the fundamental frequency of the sine wave. **b** SPWM waveform with pulse width modulation (PWM), called SPWM-PWM. The random bits are [1, 1, 1, 0, 0, 1, 1, 0, 0, 1, 0, 1] in this example. **c** Three-phase four-wire DC/AC inverter with LCL filter suitable for talkative power conversion. In this multiple-input multiple-output scenario, data streams transmitted via the three legs (marked by blue symbols) can be recovered based on measurements of the three load voltages (marked by red symbols).

Sinusoidal PWM (SPWM) is an alternative solution. With SPWM, power and data can be simultaneously and independently transmitted in each phase. For illustration purposes, focus is here on a single phase. Figure 4a depicts the conventional case without data modulation. One pulse with a time-varying duty cycle is assigned to each switching period. In conjunction with TPC, the pulses are modified to offer additional degrees of freedom to embed data. Modifications include the pulse width, the pulse position, and the pulse frequency. We call the corresponding modulation schemes SPWM-PWM, SPWM-PPM, and SPWM-FSK. Variations of TPC-based SPWM have recently been published in refs. ^52^ and ^91^. As an unpublished example, SPWM-PWM is shown in Fig. 4b. Data bits “0” and “1” are assigned to triangular carriers with different amplitudes. For bit “0,” the amplitude of the carrier is decreased compared to Fig. 4a, while for bit “1,” it is increased. Hence, the instantaneous pulse width as well as ripple fluctuations are data-dependent: a smaller/larger amplitude causes a wider/narrower pulse. The ratio between the amplitudes of the carrier signal must be balanced so that the average duty cycle (at a fixed reference voltage) is constant. This constraint is necessary to control the output voltage. The detector evaluates the data-dependent instantaneous amplitude changes of the ripple voltage. If the amplitudes differed more than shown in this example, the pulse widths between Fig. 4a, b would be easier to distinguish. A generalization to higher-order modulation schemes is possible.

SPWM-based TPC can be applied to any number of phases. SPWM-based multi-phase DC/AC power conversion is an instance of MIMO processing. The elementary building block again is the half-bridge. The data rate is proportional to the number of phases times the switching frequency. Hence, for three phases, the maximum data rate of binary SPWM-PWM and SPWM-PPM is 3*f*_sw_ bits per second, where *f*_sw_ is the switching frequency. Notably, the maximum data rate is not constrained by the fundamental frequency of the sinusoidal wave.

Given a three-phase four-wire DC/AC inverter, the voltage measurements required for data detection can be sampled on the load side (Fig. 4c). The independent data streams are only detectable, if the number of observed voltages (i.e., the number of receiver ports) is not less than the number of phases.

It is worth mentioning that the output voltage of each phase of the multi-phase inverter can again be calculated using Eq. (2) in conjunction with Eq. (1), because these equations are valid for arbitrary switching patterns, in particular also for modulated SPWM. The possibility of a time-varying duty cycle is inherent in the sampling values *s*[*n*].

### Multi-input, multi-level converters, and multiport converters

**Fig. 5:**
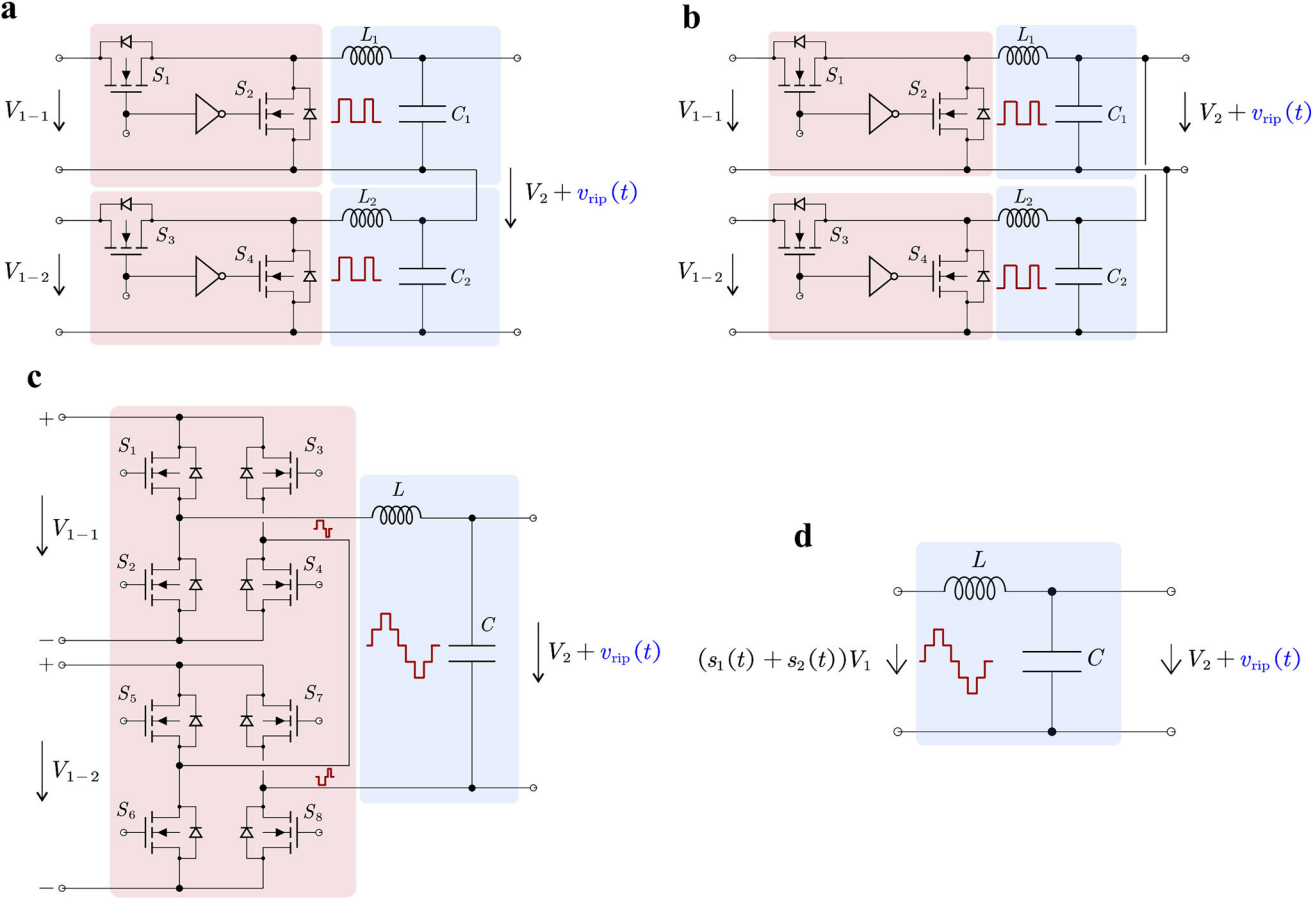
Examples of multi-input and multi-level converters. **a** Series combination of two converters. **b** Parallel combination of two converters. **c** Cascaded H-bridge multi-level converter. **d** Equivalent model of (**c**).

Power converters driven by multiple power sources are known as multi-input converters^92^. In terms of elementary combinations, in Fig. 5a, b, two fundamental examples are illustrated: a series combination and a parallel combination of two (or more) converters. The series combination is the third combination under investigation, while the half bridge is again the elementary building block. Figure 5a, b indicates that the elementary combinations are not just applicable to the half-bridge, but also to modules. In **a**, two buck converters are connected in series while in **b** they are connected in parallel. Many other topologies exist. For the series connection in **a**, a boost converter makes more sense, but we do not want to switch between different converter types. The main message is: multi-input converter topologies are multi-bit architectures from a communications point of view. When compared to single-input converters, there are more degrees of freedom for information transfer because of the multiple switching signals. Multiple ripple voltage levels can be generated and used for TPC, much like multi-level converters.

In a series combination, the output voltage is the sum of the output voltages of the components. This includes the ripple voltage. Hence, Eqs. (1) and (2) can be reapplied by adding a summation. Corresponding modeling is similar to direct-sequence code-division multiple access^93^. Without channel coding^94,95^, it is difficult or even impossible to recover all data bits at the receiver side. Besides coding, interference cancellation is recommended.

A multi-level converter (MLC) is a class of power converter topologies that can supply multiple voltage levels^81,96–99^. As an extension to conventional two-level voltage-source converters, the number of voltage levels can be any integer greater than two. MLCs are often employed as DC/AC inverters in order to attain a finer granularity of the SPWM waveform prior to filtering^96–98^. The more levels, the finer the granularity and the less filtering effort is required. Alternatively, MLCs can produce a high output voltage from sources of lower voltage^100^. The cascaded H-bridge converter (CHB)^101^, the neutral-point-clamped converter^102^, and the flying capacitor converter^103^ are the three most popular MLC topologies. The neutral-point-clamped multi-level converter, for example, consists of half bridges connected in series. Multi-level-modular converters^104^ are a widely used extension, among many others^81^. Applications that require high voltage and/or high power can be handled well with this topology^100,105^. CHB converters are suitable for a variety of applications, including devices for absorbing reactive current, adaptive power filters, solid state transformers, inverters for renewable power (photovoltaic and storage), and power electronics for transportation.

Regarding TPC, an MLC is a multi-bit architecture offering at least two benefits: (i) It is possible to generate several ripple-voltage levels. Therefore, the cardinality of the symbol alphabet can be boosted in order to increase the data rate. A CHB converter demonstrates this point (Fig. 5c). Many generalizations are possible, including CHB converters with multiple inputs; (ii) It is expected that the huge wiring effort for monitoring and control in multi-level-modular converter topologies can be avoided by TPC. In Fig. 5c, the middle points of the half-bridges are connected. Cascading is the fourth elementary combination under investigation.

The topologies discussed so far in this section can be seen as special cases of a multiport converter, and therefore, the MIMO-TPC concept can be applied to multiport converters as well^55^. Multiport architectures like the triple-active bridge and the quad-active bridge are generalizations of the DAB converter^106^. A multiport power converter is a flexible PEC with multiple ports that can be software-controlled to act as input or output ports. This converter topology is able to connect energy sources, electric storage systems, and loads. Each port has its own switching unit, often an H-bridge. Typically, all H-bridges are interconnected through a multi-winding high-frequency transformer. Multi-directional power flow is possible by adjusting the phases of the switching signals. In conjunction with TPC, data can be transmitted via any port, also simultaneously. A central controller manages energy flow and is able to provide coordinated data transmission, for example, spatial multiplexing.

Let us finally calculate the data-dependent output voltage of the CHB initially with *N* = 2 cascaded H-bridges (Fig. 5c). Similarly to the DAB converter, the differences of the switching signals between the two legs of the H-bridges are denoted as *s*_1_(*t*)∈{−1, 0, 1} and *s*_2_(*t*)∈{−1, 0, 1}, respectively. Then, for the same assumptions mentioned in the previous sections and assuming that *V*_1*−*1_ and *V*_1*−*2_ are equal to *V*_1_, the following recurrence relations can be derived:

5 $${i}_{{{\rm{L}}}}[n]={i}_{{{\rm{L}}}}[n-1]+\frac{\Delta t}{L}\underbrace{\left(\underbrace{({s}_{1}[n-1]+{s}_{2}[n-1]){V}_{1}}_{{v}_{1}[n-1]}-{v}_{2}[n-1]\right)}_{{v}_{{{\rm{L}}}}[n-1]}$$

6 $${v}_{2}[n]={v}_{2}[n-1]+\frac{\Delta t}{C}\underbrace{\left({i}_{{{\rm{L}}}}[n-1]-\underbrace{\frac{{v}_{2}[n-1]}{{R}_{{{\rm{L}}}}}}_{{i}_{{{\rm{R}}}}[n-1]}\right)}_{{i}_{{{\rm{C}}}}[n-1]}.$$

Note the striking similarity between Eqs. (1)-(2), (3)-(4) and (5)-(6) for different PEC topologies. Regarding power electronics, the waveforms can be utilized for circuit development. The equivalent model is shown in Fig. 5d, where the input voltage is (*s*_1_(*t*)+ *s*_2_(*t*)) *V*_1_. For *N* cascaded H-bridges, accordingly, the input voltage is (*s*_1_(*t*) + *s*_2_(*t*) + · · · + *s*_*N*_ (*t*)) *V*_1_. Then, the number of elements in *s*(*t*) is 2*N* + 1, i.e., the cardinality of the symbol alphabet can be made very large for the CHB-MLC converter. Therefore, the data rate can be improved by this topology. However, restoring the data bits is challenging for high-order symbol alphabets. This typically requires channel coding as discussed below.

### Outlook and research frontiers

MIMO techniques that are used for the simultaneous transmission of information and energy according to the concept of TPC are referred to as MIMO-TPC. In this Perspective, the MIMO-TPC concept is presented and elaborated for several switched-mode power converter topologies. MIMO-TPC scenarios are described by an elementary building block, namely the half-bridge, in conjunction with elementary combinations: parallel combination, interleaved combination, series combination and cascaded combination. Common to all scenarios is that the degrees of freedom for the design of communication signals are increased compared to a buck converter and related simple converter topologies employing a single switching signal. The data-dependent ripple voltage and the inductor current are numerically calculated for three selected power converter topologies. As the numerical representation is strikingly similar for different PEC topologies, a common framework for data encoding and decoding is suggested. Potential advantages of MIMO-TPC are highlighted, all of which benefit from the increased degrees of freedom for data modulation due to MIMO signal processing without compromising the efficiency of power transfer. A wide range of possible applications is anticipated. Additional MIMO-TPC topologies and modulation schemes, computer simulations, in-depth analysis, and experimental verification is stipulated for future work. The ultimate challenge is ubiquitous energy and data access.

### Open challenges and future research

Among the bottlenecks of first-generation TPC systems are limited data rates, short communication distances, and restricted networking capabilities. MIMO-TPC is expected to partly mitigate these challenges. The clue is to explore more degrees of freedom for data transmission compared to power converter topologies that use only a single switching signal, such as buck, boost, and buck-boost converters^107–110^. In the previous section, for each approach under investigation, it is outlined which degree of freedom is used to synthesize the modulated signal and to what extent each approach improves compared to SISO-TPC. For future work, the objective is to develop an evolutionary path of different MIMO-TPC architectures and to understand how each one attempts to address emerging improvements and limitations. Towards this goal, more research and experimental verification is required, see Box 1.

In recent years, the number of conceptual papers on TPC has steadily increased. Most articles published in PE journals provide experimental proof of concept. However, the first industrial products employing TPC are still lacking.

The extension of SISO-TPC to MIMO-TPC is expected to be the next logical step. If, as expected, MIMO-TPC succeeds in reducing the aforementioned bottlenecks, an expansion of the product range can be expected. However, this is an open challenge and requires further research that includes the investigation of MIMO-TPC topologies, modulation and coding techniques, receiver design, computer simulations, in-depth analyses and experimental verification.

The ultimate challenge is, according to the authors, ubiquitous energy and data access—either wireless, wired or hybrid. Communication needs energy, and energy flow optimization needs communication. Networking and multiuser communication are important steps in this direction. With state-of-the-art solutions, TPC is currently more suitable for communication between two nodes and less for large-distance communication. It is promising to apply insights from MIMO-TPC accordingly.

Machine learning and artificial intelligence (AI) are expected to considerably improve TPC systems. Initially, machine learning-based transmitter optimization and improved data detection have been identified as possible applications^111^. MIMO-TPC is particularly interesting in this context due to the large number of parameters and degrees of freedom. Transmitters should be adjusted in real time to counteract the load- and channel-dependent nature of the ripple, thereby improving energy efficiency, power quality, data rates, and reliability. Machine learning-assisted receivers are expected to be well-suited for the complex task of joint load and channel estimation, which is crucial for reliable data detection in difficult environments. At the grid level, AI may improve TPC networks by detecting anomalies and optimizing energy management. In addition, AI may support predictive network operation, improving efficiency and robustness.

Another research direction is the design of ultra-fast switches. Ultra-fast switches are an important recipe of TPC and MIMO-TPC, as the data rate is proportional to the switching frequency. A further advantage of fast switching frequencies is that the inductances and capacitances of the LC filter (Fig. 1) can be made smaller, which reduces volume and cost. In materials science, much effort is currently spent on wide-bandgap semiconductor materials like GaN. Since in MIMO-TPC even more switches and more switching patterns are applied compared to conventional SISO-TPC, ultra-fast switching is even more valuable.

Box 1 Challenges and roadmap for possible future research

- Limited data rate: The data rate can be increased by transmitting several data sequences in parallel (examples: multi-phase inverter and multiport converter) or by increasing the cardinality of the symbol alphabet (examples: interleaved converter, and multi-level converter). The latency typically reduces with increasing data rate. Among the challenges are coordinated waveform design and suitable receiver architectures. Although a common framework for data encoding and decoding is anticipated, suitable switching pattern generation is subject to upcoming research.
- Limited range, poor bit error rate: The challenge of a limited communication range is caused by the trade-off between ripple voltage and power quality. With increasing communication distance, the signal-to-noise ratio drops and therefore the bit error rate degrades. A promising but yet unexplored solution is TPC-based relaying between distant but connected power converters. Relaying is a spatial MIMO technique as several transmitters and receivers are involved. Another promising solution are modern channel coding techniques^94,95^, possibly in combination with data modulation, rate adaptation, and unequal error protection. Corresponding research in the field of SISO-TPC is still in its infancy^58^. This applies all the more to MIMO-TPC, because more opportunities are to be expected. It is worth investigating all inverter topologies, possibly using a common framework for encoding and decoding.
- Limited number of users: Networking aspects are important for bus systems and meshed networks. MIMO-based multiuser access is a powerful tool in wireless communications^93^. Likewise, in MIMO-TPC, sophisticated multiuser strategies should be explored as well. Multiport converters may serve as a good starting point.
- Power quality: A commonly noted issue with SISO-TPC is the rise in ripple voltage, which results in a degradation of power quality. Nevertheless, well-designed MIMO systems are anticipated to have the potential not just to adopt, but even to reduce ripple^112^. It is highly recommended to conduct an investigation of coded MIMO-TPC systems with the goal of enhancing power quality.

## Data Availability

Data supporting the findings of this study are available upon reasonable request from the corresponding author.
